# Medical Marijuana and the Treatment of Post Traumatic Stress Disorder: A Survey of Michigan Psychiatrists’ Opinions

**DOI:** 10.51894/001c.5117

**Published:** 2017-02-02

**Authors:** Jason Custodia, Jed Magen, William D. Corser

**Affiliations:** 1 Veteran’s Administration Los Angeles Healthcare System Los Angeles, CA; 2 Department of Psychiatry, MSU College of Osteopathic Medicine, East Lansing, MI; Statewide Campus System, MSU College of Osteopathic Medicine, East Lansing, MI; 3 Statewide Campus System, MSU College of Osteopathic Medicine, East Lansing, MI

**Keywords:** symptom relief, post-traumatic stress disorder, medical marijuana

## Abstract

**CONTEXT:**

Although recent studies have indicated a potential mechanism of action through which medical marijuana (MM) and its derivatives may treat Posttraumatic Stress Disorder (PTSD) symptoms, definitive evidence is still lacking. Few studies concerning physician attitudes regarding MM and/or marijuana-derived medications for PTSD are found in the psychiatric literature.

**METHODS:**

A non-probability convenience sample of psychiatric physicians in Michigan was surveyed during 2016. The 12-item survey questionnaire asked respondents a series of questions about their personal characteristics, prior experiences of treating PTSD and opinions concerning the use/potential use of MM for treatment of PTSD.

**RESULTS:**

A total of 83 psychiatrists (11.7% of total invited) responded to the survey. Several statistically significant correlations between respondent characteristics and other key measures (e.g., Age Category, Gender, Years of Psychiatric Practice, Psychiatric Practice Role (i.e., resident vs. attending), Number of Psychiatric Subspecialties, and Number of PTSD Patients Diagnosed and/or Treated to date) were found. A composite summary score was also formulated from questions related to opinion regarding the use of MM for PTSD and categorized into three comparison groups. The final stepwise multinomial logistic model demonstrated three statistically significant factors influencing what response category respondents fell into regarding MM use for PTSD: a) how often respondents had been exposed to recommendations concerning the use of MM for PTSD (p < 0.001), b) Age Category (p = 0.001) and how frequently respondents had recommended MM for treatment of PTSD (p < 0.001).

**CONCLUSIONS:**

The results from this smaller sample indicate that the majority of psychiatrist respondents did not support MM for the treatment of PTSD. Judging from these results, Michigan psychiatrists may be extremely conservative regard the prospective use of MM for PTSD. Few sample respondents indicated that they had been exposed to professional literature detailing MM and derivatives as a treatment for PTSD. Most respondents also indicated that they were *Unsure/There is Not Enough Research* concerning the scientific evidence for the use of MM for PTSD. Based on these findings from a smaller sample, the use of MM and its derivatives for treatment of PTSD may not currently be supported by the majority of Michigan psychiatrists.

## INTRODUCTION

Post-traumatic Stress Disorder (PTSD) is a psychiatric condition resulting from having experienced or witnessed a traumatic event, with a characteristic constellation of symptoms including event re-experiencing, avoidance of event reminders, and hyper-arousal.^1^ Although the great majority of people who experience a traumatic event never develop PTSD, some may develop long-lasting symptoms which can greatly affect their daily functioning and overall quality of life.

According to the U.S. National Institute of Mental Health’s most recent statistics, PTSD has a lifetime prevalence of 6.8%, with a 12-month prevalence of 3.5%. Of those cases, over a third are classified as *severe*.^2^ Although PTSD is among the most debilitating and prevalent of psychiatric conditions, it remains among the most recalcitrant conditions to treat with highly variable prognoses.^3^ Indeed, some patients suffer from reemergence of symptoms in spite of prolonged treatment.^3^

Clinicians’ typical difficulty in ameliorating the symptoms of PTSD has driven a search for potential therapeutic agents that may provide relief.^3^ For many patients afflicted with PTSD, the only agent they feel can effectively palliate their symptoms may be medical marijuana (MM).^4^ Although marijuana has been listed as a Schedule I controlled substance since the inception of the *Controlled Substances Act of 1970*,^5^ there has been a growing movement to approve its use as a specific therapeutic agent for PTSD. In seven states and the District of Columbia, PTSD is now an approved condition for the prescribed use of MM.^6^

In 2014, the State of Michigan legislature approved the therapeutic use of MM for PTSD after a panel of non-physician experts heard patients’ testimony.^7^ However, these MM use policy changes came with little input from physicians, and virtually none from psychiatrists, those clinicians most likely to treat PTSD. Currently, national psychiatric PTSD treatment guidelines do not universally advocate including the therapeutic use of marijuana or any marijuana-derived medications.^3,8^ In fact, the most recent practice 2009 guideline update from the American Psychiatric Association makes no mention of marijuana as a possible treatment option for PTSD.^8^

Due to MM’s conflicting legal status (i.e., being legal at the state level but illegal at the federal level), very few, if any, physicians now appear to prescribe marijuana in any form to patients.^4^ Although studies concerning the effects of marijuana and its derivatives have steadily increased during the past decade, there are still only a handful of published studies concerning the effect of MM on PTSD symptoms.^8^

### PTSD and Marijuana Use

There has long been an anecdotal association between the use of marijuana and PTSD symptoms.^4^ Most studies to date have focused on the well-known negative consequences of cannabis use, including an increased risk for several other substance use disorders.^4^ However, other groups have sought to specifically examine the relationship between PTSD symptoms and MM use.

For example, a study in 2011 by Cougle et. al., determined that having a diagnosis of PTSD significantly increased the odds of a lifetime patient history of cannabis use, with 50% of sample subjects stating that their PTSD symptoms occurred prior to, or around, the same time as their first cannabis use.^9^ A similar follow-on study demonstrated that cannabis users with higher PTSD scores were significantly more likely to use cannabis to improve their sleep and coping than subjects with lower PTSD scores.^10^ The results from these initial studies have suggested that MM may exert some neurobiological effect resulting in perceived relief of PTSD symptoms.

### Neurobiological basis for the use of marijuana in PTSD

Within the past five years, more critical reviews of the medical literature regarding the use of marijuana for PTSD symptoms have been published. These reviews have cited many of the findings already described in this paper, but have also provided some notable new insights. Two separate reviews detailed marijuana’s possible mechanism of action through the effects of delta-9-tetrhydrocannabinol (THC), the main psychoactive cannabinoid in marijuana, on endogenous cannabinoid receptors of the brain’s endocannabinoid system (ECS).^11,12^ The ECS appears to be involved in memory formation, fear, and emotion or executive functioning, areas of the brain likely active during memory formation/extinction in PTSD.^11,12^

Other studies have focused on the effects of marijuana-derived medications on the ECS, including the FDA-approved medication Dronabinol ^13^ (i.e., indicated for appetite stimulation in cancer patients and anorexia), the synthetic cannabinoid Nabilone ^14^ (i.e., primarily used as an antiemetic for cancer), and the endogenous cannabinoid neurotransmitter Anandamide (for anxiety/depression).^15^ Some research has focused on imaging studies using positron emission tomography (PET) to examine the ECS, showing an increase in the concentration of cannabinoid receptor availability in subjects with PTSD versus healthy controls.^16^

However, there is still a dearth of information about psychiatrists’ opinions on this topic. Only one survey project was found by the authors during the past 20 years that attempted to gauge physicians’ opinions on the use of MM for any indication.^17^ The results from this project indicated that physicians were, in general, less supportive than the general public regarding the use of MM.^17^

In summary, research has indicated that the ECS appears to be a viable therapeutic target to treat PTSD, and that cannabinoids (both synthetic and endogenous) may be potential therapeutic agents. However, many of these studies used smaller samples and have not been replicated. Furthermore, it remains unknown whether these results have been routinely disseminated to practicing psychiatrists.

In light of the increasing number of states legally approving MM for PTSD, it is imperative to examine the opinions of behavioral health professionals who treat PTSD about this newer treatment modality. Since psychiatrists most frequently treat PTSD patients, their expertise is extremely important in helping shape the discourse concerning this potential PTSD treatment.

### Project Purpose

This exploratory pilot study was conducted to investigate the perspectives of psychiatrists on the evolving topic of MM use for treatment of PTSD. The authors conducted a survey of attending and resident psychiatrists in Michigan to ask respondents a number of questions related to the potential use of MM and/or its derivatives for the treatment of PTSD. A secondary goal of this study was to clarify psychiatrists’ current opinions concerning the use of MM versus marijuana-derived medications for the treatment of PTSD symptoms.

Before the study, the authors had generally hypothesized that younger (i.e., resident) psychiatrists would be more receptive to prescribing MM for PTSD. In addition, the authors had speculated that those respondents more familiar with guarded recommendations from groups (e.g., American Academy of Neurology,^18^ Federation of State Medical Boards^19^) for the use of MM to palliate PTSD symptoms would prove more supportive.

## METHODS

**Figure 1:**
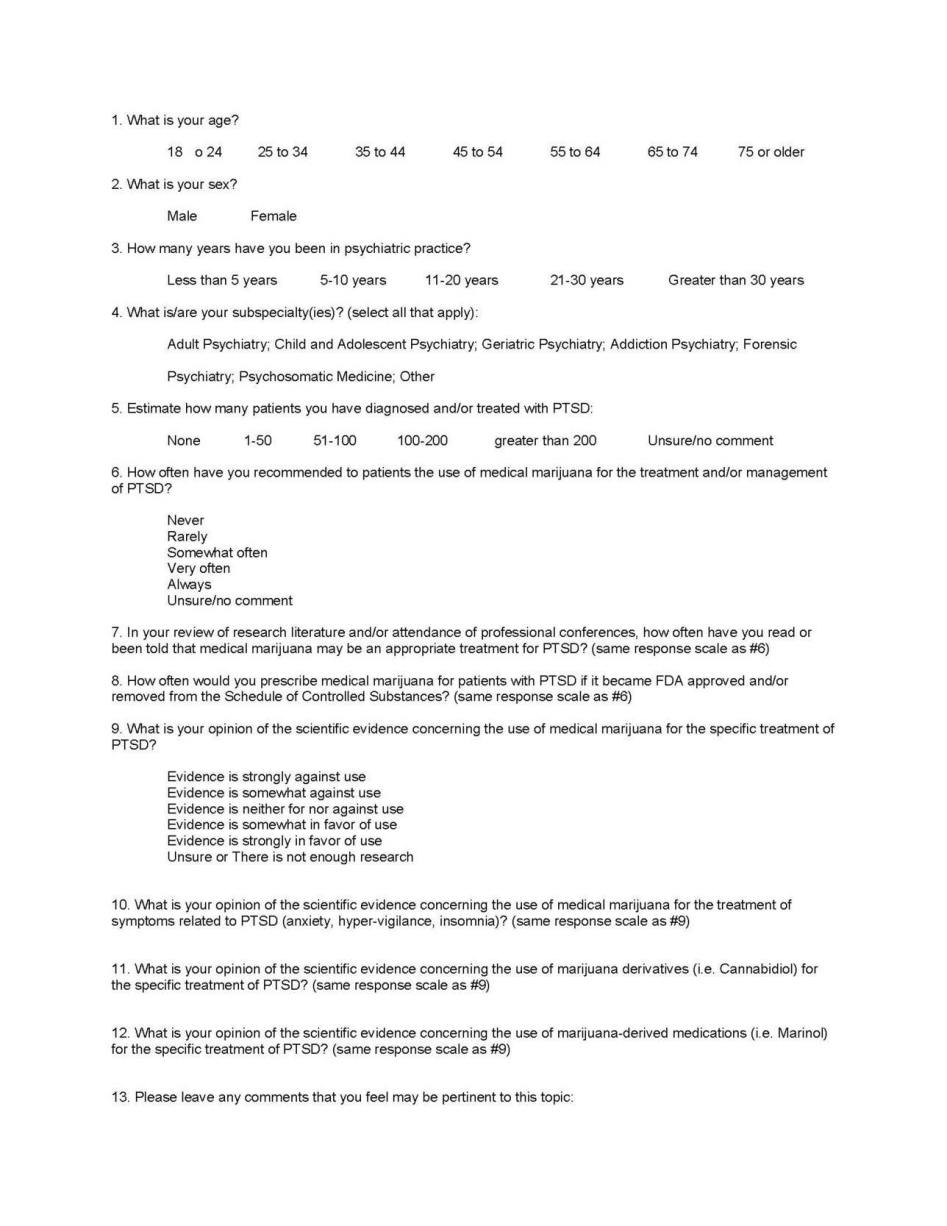
Survey Items of *Psychiatrists’ Opinions on Medical Marijuana for Post-Traumatic Stress Disorder*

A cross sectional email-based survey was distributed to a total of 723 licensed psychiatrists in the state of Michigan between February 11, 2016 and March 11, 2016 using the *Survey Monkey* internet-based survey program.^20^ The survey developed by the first two authors asked respondents a series of 12 questions regarding their professional opinions about MM and its derivatives as choices for the treatment of PTSD symptoms. Most opinion questions used a Likert-type scale, including *No Response/Unsure* response option. An additional open-ended comment item was also added at the end of the survey to further gauge psychiatrists’ opinions on the study topic (see Figure 1).

### Study Population

Subjects were drawn from an email database of psychiatrists currently practicing in the state of Michigan. The email database had been obtained from the state branch of the American Psychiatric Association, with study approval obtained through the Michigan State University institutional review board.

### Data Analyses

All data analyses were conducted using *S.P.S.S. Version* *22*.^21^ A series of descriptive statistics were first generated with cross-tabulations completed to examine for statistically significant bivariate correlations across major study measures to inform the conservative selection of later multivariate modeling procedure model terms. Key correlations between respondent characteristics (i.e., Age Category, Gender, Years of Psychiatric Practice, Psychiatric Practice Role (i.e., resident vs. attending psychiatrist), Number of Psychiatric Subspecialties, and Number of PTSD Patients Diagnosed and/or Treated to Date) were initially examined.

Finally, a forward stepwise *Main Effects* multinomial logistic regression (MLR) model comprised of potentially-significant model terms was conducted. Such a modeling procedure was more appropriate for this non-normally distributed sample to predict the probability of *use of MM for PTSD* category membership based on major respondent characteristics.^22^ In this MLR model, each model term was entered individually (i.e., *stepwise*) to gauge their significance for the three composite score categories (i.e., *Generally Against Use of MM for PTSD*, *Neither Against of Supportive* of Use of MM for PTSD and *Generally Supportive of Use of MM of PTSD*), with those variables with non-significant test statistics (i.e., greater than p value of 0.100) removed from the final predictive model.

## RESULTS

Of the 723 email surveys sent, 16 (2.2%) were not successfully delivered due to invalid email addresses. Of the 707 remaining surveys sent to active email accounts, 83 (11.7% of total invited) subjects responded to the survey. A total of 34 (41%) respondents reported being younger than 35 years of age, with the seven age categories of respondents quite diverse. A total of 44 (53.0%) respondents were males (See Table 1).

**Table 1:** Sample Respondent Characteristics** (N = 83 Psychiatric Physicians)

	n	% of category
1. Age Category *		
18 to 34 Years Old	34	41.5
35 to 54 Years Old	29	35.4
55 Years and Older	19	22.9
2. Gender *		
Male	44	53
Female	38	45.8
3. Years of Psychiatric Practice *		
Less than Five Years	42	50.6
Five to 20 Years	22	26.5
21 Years or More	18	21.7
**4. Number of Patients Diagnosed/Treated for PTSD To Date**		
None	1	1.2
Between 1 and 100 Patients	48	57.8
Greater than 100 Patients	26	31.3
Missing	8	9.6

* Data from One Respondent Missing

A total of 42 respondents (50.6%) reported having practiced in psychiatry for less than five years at time of survey. The average number of psychiatric subspecialties reportedly held by respondents was 1.05 (SD 0.825). Forty-eight (57.8%) sample respondents reported having diagnosed or treated a broad range of between 1 and 100 PTSD patients at time of survey, with 26 (31.3%) additional respondents indicating that they had treated more than 100 PTSD patients (See Table 1).

Notably, only one respondent (1.2%) indicated that they *Sometimes* *recommended the use of MM for PTSD*, with the great majority of the remaining sample answering *Never* (90.4%). Only 11 (13.3%) of total respondents indicated that they had *Sometimes* or *Often* been exposed to the notion of MM being an appropriate PTSD treatment in the professional literature or at conferences.

Thirty-one respondents (37.4%) stated they would *Never* prescribe MM, even if it became FDA approved and/or removed from the federal *Schedule of Controlled Substances*. Twenty-five other respondents (30%) said they would *Rarely* prescribe MM for PTSD, with 17 of remaining respondents (20.5%) surveying that they were *Unsure/No Comment*. In the survey items related to respondents’ opinions on the actual scientific evidence of MM for the treatment of PTSD (Questions 9-12 in Figure 1), a majority of the respondents (n = 44) selected the answer *Unsure/There is Not Enough Research*.

### Key (significant and non-significant) bivariate correlations included:

1. Age Category with a) Gender (p = 0.030, with women respondents tending to be younger), and b) Category of Number of PTSD Patients Diagnosed/treated to date (p < 0.001, with older respondents having treated more PTSD patients);
2. Years of Psychiatric Practice with likelihood category of having recommended MM to patients more frequently in the past (p = < 0.001), with more experienced respondents more likely to recommend MM for PTSD); and
3. More frequent exposures to recommendations concerning use of MM and composite MM use belief score category (p = 0.001, with more frequently-exposed respondents more likely to prescribe).

The survey included three different related questions related to respondents’ opinions regarding use of MM to treat PTSD which were conservatively categorized into three overall groups: 1. *Neither Against or Supportive of MM use for PTSD*, (n = 11, 13.3%) 2. G*enerally Against MM use for PTSD*, (n = 19, 22.9%) and 3. *Generally Supportive of MM use* *for PTSD* (n = 9, 10.8%). It should be acknowledged that 44 respondents (53.0%) opted to not answer at least one of these three survey items. This overall opinion categorical measure was treated as a de facto *outcome measure* for most subsequent analyses.

**Figure 2:**
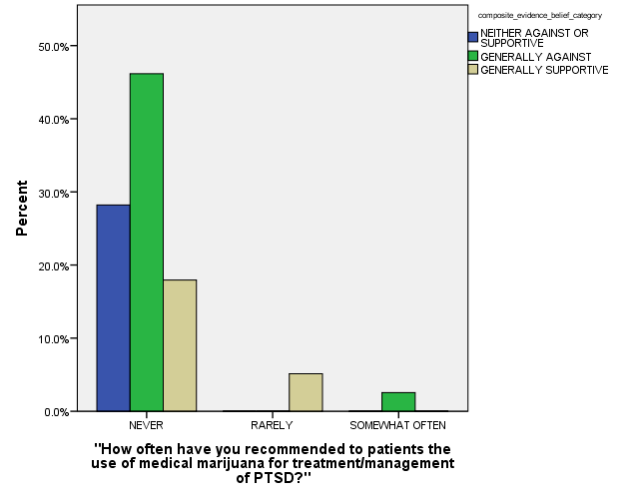
Composite Opinion Categories regarding the Use of Medical Marijuana for Post-Traumatic Stress Disorder** (n = 39)

**Figure 3:**
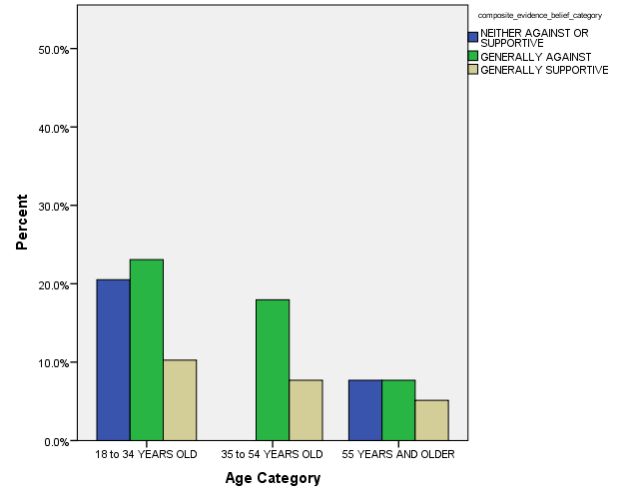
Composite Belief Category for use of Medical Marijuana for Post-Traumatic Stress Disorder by Age Category** (n = 39)

**Figure 4:**
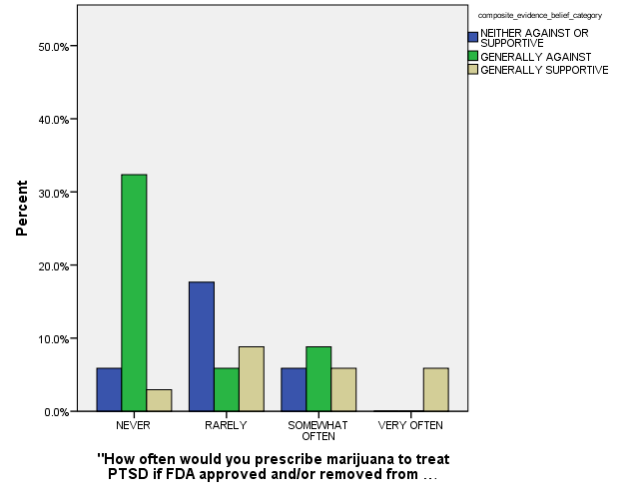
Composite Belief Categories by Likelihood of Future Prescription of Medical Marijuana for Post-Traumatic Stress Disorder (if ever FDA Approved)** (n = 39)

In the final MLR stepwise model for outcome, the following model terms remained statistically significant after controlling for other non-significant model terms: a) How Often Respondent had been Exposed to Recommendations concerning use of MM for PTSD patients (p < 0.001), b) Age Category (p = 0.001), and c) How often Respondent had Previously Prescribed MM for PTSD (p < 0.001) (see Figures 2, 3, and 4 for general depiction of key results). The *goodness of fit* and other model fitting information from the final MLM model were each quite adequate, although the number of respondents with entirely complete survey data that could be included into this stepwise model was especially low (n = 27). Still, the authors were able to identify these statistically significant predictors of respondents’ opinions regarding MM use for PTSD in spite of their limited sample size.

### Limitations

This study did have several constraints related to the inherent limitations of smaller cross-sectional survey studies. The results may have also been skewed by other unmeasured factors, including a *self-selected* group of psychiatrists actually responding to the survey. For instance, respondents in this sample were predominantly young and less experienced, with approximately 40% in the 25-34 *years* age group category and over half of the respondents in psychiatric practice less than five years.

Based on fairly loose national estimates, it is certainly possible that these respondents comprised a somewhat non-representative (i.e., younger and less-experienced than average) group of Michigan psychiatrists.^23^ As for the survey tool itself, the questionnaire had not been psychometrically tested before the study, having only been previously examined by several psychiatric experts for overall validity. During these initial survey reviews, the clarity of some of the questions wording and use of terms and response options had been edited.

## CONCLUSIONS

The results of this initial pilot study suggest that while a majority of the psychiatrists in the sample were treating PTSD patients at time of survey, they remained quite conservative in regard to using MM to alleviate patients’ PTSD symptoms. This is evidenced by that fact that only one (1.2%) of the 83 respondents stated they had actually recommended the use of MM for the PTSD treatment in the past. Also, a large majority of psychiatrist respondents stated that they would not prescribe MM even if it were to be taken off the FDA’s list of controlled substances. The authors’ hypotheses that both younger respondents and those more familiar with established guidelines for MM use would be more supportive of MM use were not generally supported by results.

These results also demonstrate that few psychiatrists may be getting exposed to recommendations in the professional literature or conferences regarding MM and/or its derivatives as a treatment modality for PTSD. Only 11 (13.3%) of the total sample reported that they had *Sometimes* or *Often* been exposed to such recommendations. This is in line with responses made related to the actual perceived scientific evidence for the use of MM for PTSD, in which a majority of the respondents indicated *Unsure/There is Not Enough Research*. Even though the authors’ final MLR stepwise model showed three statistically significant model terms related to respondents’ categorical opinions in the area, it is difficult to draw definitive conclusions from these study findings.

Perhaps the most revealing study finding came from the comments section in which respondents were allowed to provide open-ended statements concerning this practice issue. Many of these qualitative responses expressed ardent positions against the future use of MM for PTSD symptoms, but also against marijuana in general. As one psychiatrist stated, *I have seen enough functional deficits from marijuana in patients across all age spectrums as well as cognitive decline.* *I would never prescribe medical marijuana for any reason.* Many other respondents pointed out the negative effects of medical marijuana as a barrier to its therapeutic use.

Another respondent also questioned the validity of legislative processes for MM approval, stating, *Medical marijuana is a lie. It was passed in several states by vote or legislative [action] rather than scientific rigor like other medicines. There are many cannabinoids and some may be useful but we need more studies.*

In fact, the call for more research and literature on MM was a common theme amongst respondent comments. For example, one respondent stated, *Thank you for including me in this survey. Some of your questions are quite provocative and will cause me to pursue additional reading in this area. It would be helpful if you provided your respondents a few relevant citations on the subject.*

Future studies in this area would benefit from the use of psychometrically-tested surveys lending themselves to more detailed statistical analyses. It should be clearly acknowledged that this project team was very likely underpowered to detect meaningful sample subgroup differences that might be otherwise identified within larger future samples. Since marijuana is still illegal under federal law, doctors may not technically prescribe MM without violating the law, even in states where MM agents have been approved. Therefore, it is not unreasonable to conclude this fact could have influenced the responses from this study sample.

However, this cross-sectional survey study is still apparently one of the first to systematically examine the opinions of psychiatrists on the use of MM for the treatment of PTSD. Judging from these results, this remains an especially complex clinical care issue, with therapeutic, social, and legal facets that have not yet been thoroughly considered by practicing psychiatrists. As such, there is still a vital need for more studies to further clarify clinician opinions concerning the potential use of MM and marijuana-based medications for this disorder.

There remains a paucity of results from randomized controlled studies that could be used to inform current clinical guidance in the use of MM for PTSD. Ideally, the results from future research can inform the development of more evidenced-based guidelines for practicing psychiatrists.^6,8,9^

### Conflict of Interest

The authors declare no conflict of interest.
